# The Nest Architecture of the Ant *Odontomachus brunneus*

**DOI:** 10.1673/031.010.6401

**Published:** 2010-06-14

**Authors:** Lina M. Cerquera, Walter R. Tschinkel

**Affiliations:** Department of Biological Science, Florida State University, Tallahassee, FL 32306-4370, USA

**Keywords:** casting, colony size, head width, trap-jaw ant, worker number

## Abstract

The architecture of the subterranean nests of the ant *Odontomachus brunneus* (Patton) (Hymenoptera: Formicidae) was studied by means of casts with dental plaster or molten metal. The entombed ants were later recovered by dissolution of plaster casts in hot running water. *O. brunneus* excavates simple nests, each consisting of a single, vertical shaft connecting more or less horizontal, simple chambers. Nests contained between 11 and 177 workers, from 2 to 17 chambers, and 28 to 340 cm^2^ of chamber floor space and reached a maximum depth of 18 to 184 cm. All components of nest size increased simultaneously during nest enlargement, number of chambers, mean chamber size, and nest depth, making the nest shape (proportions) relatively size-independent. Regardless of nest size, all nests had approximately 2 cm^2^ of chamber floor space per worker. Chambers were closer together near the top and the bottom of the nest than in the middle, and total chamber area was greater near the bottom. Colonies occasionally incorporated cavities made by other animals into their nests.

## Introduction

The superorganism metaphor suggests that the subterranean nest a colony of ants constructs should be regarded as a functional part of the colony superorganism. The particular architecture of the nests of different species can be hypothesized to serve superorganismal functions in particular ways suited to the biology of each species. The study of nest architecture can therefore potentially lead to important understanding about how ant colonies work. Unfortunately, the study of subterranean ant-nest architecture is in its infancy. Although a few descriptive studies have begun to outline the range of architectural variation within and among species (reviewed by Tschinkel 2004a, 2004b), understanding of the functional aspects of this variation is far in the future. The situation has recently improved, but most reports have provided only verbal descriptions or simple drawings based on excavations, and very few included a census of the colony or quantitative details of the architecture. The architecture of the nests of the fungus-gardening ants has received more attention than that of most other groups (Jonkman 1980a, 1980b; Moreira et al. 1995, 2004a, 2004b; Mueller and Wcislo 1998; Solomon et al. 2004; Fernández-Marín et al. 2005; Klingenberg et al. 2006; Verza et al. 2007).

Nevertheless, ants clearly excavate species-typical subterranean nests, a conclusion strengthened by the more recent work of Tschinkel (1987, 1999, 2003, 2004b, 2005), Mikheyev and Tschinkel (2004), and others (Ruano and Tinaut 1993; Plaza and Tinaut 1989; Moreira et al. 2004a, 2004b; Forti et al. 2007). Despite an enormous range of size, a large proportion of ant nests are composed of two basic elements, more or less vertical shafts connecting horizontal chambers (Tschinkel 2003). The architectural variation among species is largely the result of variation in the form, spacing, and size of these elements. Nests with similar architecture can vary in depth from a few centimeters to 4 m or more (Tschinkel 2003). Because nest excavation is a group activity, the manner in which the architecture results from self-organized behavior has stimulated experimental and modeling analysis of ant tunneling activity (Buhl et al. 2006; Rasse and Deneubourg 2001). Gas gradients in ant nests have been modeled because they have been suggested as templates for nest construction (Cox and Blanchard 2000; Tschinkel 2004b). New study methods include x-ray computed tomography, which has been applied to the study of the growth of small Argentine ant nests in the laboratory (Halley et al. 2005). Trace fossils interpreted as having been constructed by ants have also received considerable interest (for a review, see Hasiotis 2003).

As in any young field, however, the structure and range of variation of the nests of a variety of ant species must be described in quantitative terms, as must the distribution of the ants within these structures given that the road to the universal leads via the particular. The present paper provides a description of the nest architecture and its variation for the ant, *Odontomachus brunneus* (Patton) (Hymenoptera: Formicidae), and together with several previous papers (Tschinkel, 1987, 1999, 2003, 2004; Mikheyev and Tschinkel, 2003), contributes to the beginnings of a systematic study of ant-nest architecture for its own sake.

## Materials and Methods

### The study site

All nests of *O. brunneus* studied were located in an area of sandhills longleaf pine forest 3.2 km southeast of the Tallahassee Regional Airport (30° 37′ 60″ N, 84° 32′ 28″ W). The site has a relief of about 10 m; excessively drained, deep sandy soils; and a forest of longleaf pine and turkey oak. The ground cover consisted of sparse wiregrass, shiny blueberry, scattered palmetto, other small shrubs, and scattered leaf-litter patches. The study spanned from August to December 2007.

### Plaster casting and excavation

Nests of *O. brunneus* were initially located by the characteristic soil depots around the entrance. Identity was confirmed by collection of ants emanating from the nest. For casting, orthodontic plaster (Labstone, Modern Materials, http://heraeus-dental-us.com/en/ourproducts/laboratory_1/mondernmaterials/mondernmaterials_1.aspx) was mixed with water to form a very thin slurry. The nest entrance was cleared with a portable vacuum cleaner, and a small berm was constructed around it. The plaster slurry was poured directly into the entrance until the nest filled. As the soil drew water from the slurry, more plaster slurry was added to keep the nest filled. After about an hour, the plaster had set sufficiently to be excavated. A pit 0.5 to 1.5 m in depth was dug to one side of the nest, and the cast was then excavated laterally from its side, upper regions first. Casts always broke during excavation and had to be reconstructed later in the laboratory.

### Metal casting and excavation

A few nests were cast in molten aluminum or zinc. The metals were melted in a charcoal-fired kiln and poured directly into the nest entrance. The procedure is described by Tschinkel (2010). Excavation proceeded as for plaster casts. The advantage of metal casting is that the cast does not break during excavation. These casts were used as intuitive guides during reassembly of the plaster casts and to confirm their structures.

### Cast reconstruction, imaging and measurement

The cast pieces were dried and cleaned in the laboratory and the nest reassembled; 5-min epoxy was used to cement the pieces together. The completed cast was laid on a black background and photographed digitally from at least two vantages with a scale. Stereo pairs of photographs (together with a suitable viewer or ocular technique) allow viewing of the cast in three dimensions. The scale in the images allowed various aspects of the casts to be measured. After completion of the photographs, the casts were broken into chambers and connecting shafts, and the chambers photographed with a scale from directly above. Measurements of chamber dimensions and area were made from these images.

### Dissolution of the casts and census of the ants

Finally, the broken cast pieces of each nest were tied into fine-mesh fabric bags and placed in a bucket with slowly running hot water. The top, middle, and bottom thirds of the cast were bagged separately. In 3 to 4 weeks, the hot water dissolved all the plaster and left the remains of the ants in the fabric bags, along with all accompanying materials in the cast. The ant heads were separated from the debris, counted, and mounted on cards with double-stick tape for digital imaging with a scale. Head width across the eyes, head length, and head width at the narrowest part of the head were measured from these images with the included scale. Other significant materials, such as cocoons/brood or possible predators, were also examined and their distribution within the nest determined.

## Results

(Figures of casts are shown online in the Appendix “Casts of nests”.) The nests of *O. brunneus* were rather simply structured. Each consisted of a single, more or less vertical shaft connecting a varying number of chambers. Stereo images of these casts are shown in Figures 1, 2, 3, 4, 5, 6, 7, 8, 9, 10, 11, 12, 13, 14, 15, 16, 17, 18, and 19. Surfaces of most casts were fairly rough, indicating rough inner nest walls. In several nests, the ants seem to have broken into the excavations of other animals and incorporated them into their own nests. Use of plant roots was also observed. The upper region of Nest 8 was probably originally a rodent burrow, and the lumpy chambers at the bottoms of Nests 2, 10, and 15 were probably made by other animals, as were the complex tunnels in the upper parts of Nest 15.

**Figure 20.**
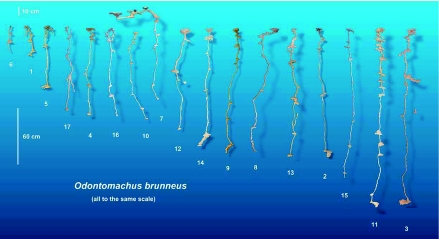
All casts shown to the same scale in order, from left to right, of increasing size and chamber number, showing the changes that occur during nest growth. High quality figures are available online.

Nests ranged greatly in size, comprising 2 to 17 chambers. Maximum depths ranged from 18 to 184 cm and total chamber area from 28 to 340 cm^2^ (Figures 3, 7). Figure 20 shows all of the casts to the same scale and illustrates the changes of nest size, shape, and composition that occur as a nest grows from small to large. In general, all elements of the nest increased simultaneously, including maximum nest depth, mean chamber area, and number of chambers, making the nest proportions (nest “shape”) relatively size-free.

**Figure 21.**
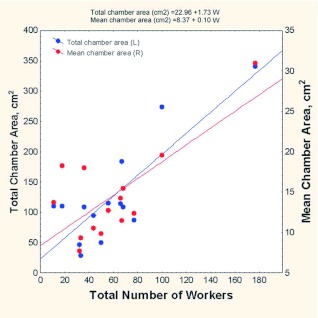
Total nest area and mean chamber size increase as the number of workers in the colony increases. Larger chambers thus reflect nest growth. High quality figures are available online.

Because the plaster casts were dissolved and the workers entombed in them censused, the worker census could be associated with nest characteristics. Not surprisingly, nest size increased with the number of workers in the colony, which ranged from 11 to 177. Each additional worker was associated with an increase in total chamber area of 1.7 cm^2^ (total chamber area = 23 + 1.72 (no. of workers); r^2^ = 0.69; p< 0.0005), and the mean chamber size increased by 0.1 cm^2^ (mean chamber area = 8.37 + 0.104 (no. of workers) (Figure 21)). The relationship between chamber area and worker number held even when the latter were vertically cumulated into top, middle, and bottom thirds of the nest. Levels with more chamber area had significantly more workers in them (number of workers in level = 3.51 + 0.38 (area in level); R^2^ = 26%; *F*_1,40_ = 15.61; p < 0.0003), as expected from the positive relationship between total chamber area and total workers.

A plot of the area per worker (not shown) revealed that this value was constant at about 2 cm^2^ per worker across most colony sizes, with the exception of two colonies with very few workers (Nos. 5 and 15, excluded from the analysis below). These colonies had probably recently lost workers rather than having excavated relatively larger nests, or perhaps workers were simply outside the nest at the time of casting. Colonies therefore seem to excavate a similar area of chamber for every worker. The dorsal silhouette of workers of *O. brunneus* measures about 7.5 mm^2^ in area if the legs, mandibles, and antennae are excluded, and about 26 mm^2^ if the lateral extension of femurs is included. The worker dorsal silhouette (without legs) therefore occupies an average of 4.8% (SD 2%, two outliers excluded) of the chamber area available per worker, and with legs 15% (SD 8%, two outliers excluded). The nest the Florida harvester ant, *P. badius*, contained a mean of 1.4 cm^2^ per worker (SD 0.74), of which worker bodies (without legs) took up about 18% (SD 8.4; unpublished data).

Nests of the ant *Camponotus socius* contained an average of 1.1 cm^2^ (SD 0.41) of floor area per worker, of which the worker body (without legs) occupied a mean of 16% (SD 5.4%; unpublished data). *P. badius* and *C. socius* are therefore about equally crowded, and both appear to be more than three times as crowded as *O. brunneus*.

**Figure 22.**
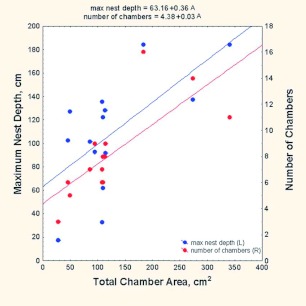
As nests grow in total area, the number of chambers and the maximum nest depth increase. Nest deepening and the addition of chambers reflect nest growth. High quality figures are available online.

Several components of nest size also increased with nest size, measured as total chamber area or total number of chambers. Averaged over all chambers, the mean chamber area was about 15 cm^2^ (SD 14.6 cm^2^), but averaged by colony, it increased with colony size (mean area = 10.7 + 0.033 (total area); r^2^ = 30%). Nests grew through deepening and the addition of more and progressively larger chambers (Figure 22). For every 100 cm^2^ increase in total chamber area, the nest was 36 cm deeper and had 3 additional chambers (max. nest depth = 63.1638 + 0.3605 (total area); number of chambers = 4.3805 + 0.0304 (total area)).

Because all of these measures were correlated with each other, other ways of describing the changes associated with nest growth are also possible. For example, the addition of each chamber increased total chamber area by about 18 cm^2^, and each additional chamber averaged about 0.6 cm^2^ larger than the previous chamber, so chambers in the smallest nests averaged about 9 cm^2^ and those in the largest about 31 cm^2^. Moreover, the addition of each chamber was associated with an increase in nest depth of 8.7 cm.

**Figure 23.**
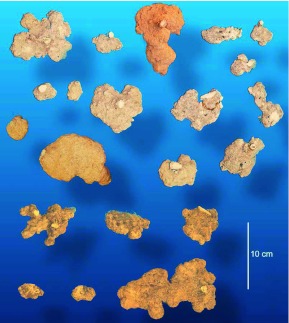
A sampling of chambers, viewed from directly above, showing the simple, roughly circular outlines of most. A few larger chambers may have more complex, lobed outlines. High quality figures are available online.

The chamber shapes ranged from nearly circular to somewhat oval or irregular (Fig. 23), but with a few exceptions (mostly the bottom chambers), they did not deviate strongly from circularity; that is, they were not strongly lobed. More than 70% of chambers had circularities greater than 0.6.

**Figure 24.**
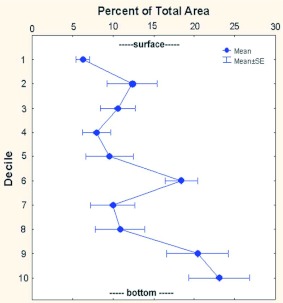
A greater proportion of total area is located near the bottom of the nest; i.e., nests are moderately bottom-heavy. “Deciles” are tenths of the maximum nest depth. High quality figures are available online.

Chamber area was not evenly vertically distributed within the nest. For comparison of nests of differing sizes, chamber area was converted to percentage of the total area and depth to deciles (1 decile = 1/10^th^ of maximum nest depth), yielding a size-free estimate of nest “shape” (Mosimann and James 1979). Figure 24 shows that, on average, a higher proportion of total area occurred at greater depth, i.e., that nests were bottom-heavy (one-way ANOVA: *F*_1,9_ = 4.91; p < 0.00002). The size-free shapes of small, medium, and large nests did not differ significantly, so only the overall average is shown in Figure 24.

**Figure 25.**
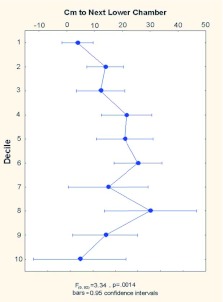
Chamber spacing is not even but is somewhat closer near the top and bottom of the nest than in the middle. “Deciles” are tenths of the maximum nest depth. High quality figures are available online.

All nests had one or more chambers near the surface and usually ended in a chamber at the bottom. The spacing between the chambers was least near the surface and near the bottom and greatest at the middle depths (one-way ANOVA: *F*_1,9_ = 3.34; p < 0.002) (Figure 25), a trend that can also be seen in the images in Figure 20. Although, during excavation, workers seemed to be more abundant in the upper and lower levels of the nest, this trend was not significant.

### Seasonal effects

Most of the nest casts were made in September and December 2007; only one each was made in October and November. Worker size, as measured by head width, was greater (1.70 mm) in the November–December nests than in the September–October nests (1.62 mm) (*t*-test: t_10_ = 2.36; p < 0.05), perhaps as a result of improved nutrition later in the season, because total number of workers did not differ. No other measure differed by season.

Worker head width, averaged by nest level, ranged from 1.53 mm to 1.8 mm and was isometric with head length (regression: HL = 0.31 + 1.02 HW; *F*_1,29_ = 260; p < 0.00001; R^2^ = 90%). Worker heads do not therefore change shape with increasing head size.

### Other nest contents

In addition to workers and their parts, the dissolved casts yielded other materials, including seeds, parts of other ant species, other insect parts, and diverse plant material, as well as cocoons and larvae. *O. brunneus* often decorates its nest crater with caterpillar frass, seeds, and other debris, but whether this tendency is biologically meaningful is unknown. The insect parts found in the casts were probably the remains of prey. Cocoon distribution in nests and throughout the season varied but did not show any clear trends.

### Discussion

No matter what their size, the nests of *O. brunneus* can be recognized by their characteristic appearance; that is, the size-free shape does not change much with nest size, as is apparent in Figure 20. This independence of size-free shape from total size is also apparent in the nests of *Pogonomyrmex badius* and *Camponotus socius* (Tschinkel 2004b, 2005) and means that workers need only follow simple, local iterative rules to produce a nest of similar shape but any size. In laboratory “sand sandwiches,” workers of *Messor sancta* excavated networks of tunnels, some features of which were invariant across network size (Buhl et al. 2006).

The nests of *O. brunneus* are simple vertical shafts connecting simple, horizontal chambers, a widespread architectural unit among subterranean ant nests. The ancestors of the ants probably dug such burrows, though probably with a single, or very few chambers. The chamber floors probably provide the work and living space, and their total area is thus proportional to the number of ants in the nest; about 2 cm^2^ of floor space is provided per worker, of which a minor fraction is actually occupied by the worker's body. Available data show that *O. brunneus* is one-third as crowded as *P. badius* and *C. socius*. Such variation among species in crowding may affect the rates of interaction among workers and could thus be used to “tune” colony functions depending on rates of interaction, but because these calculations are means for the entire nest, whereas in reality, the workers are not distributed evenly in the nest, they are often much more crowded in the lower parts of the nest (Tschinkel 1999).

All but one of the nests used in the present study were at the same location, a very dry, open, longleaf-pine forest several meters above the water table. Nests at a moister, heavily oak-shaded site near a temporary pond were considerably shallower during the summer but deepened in the winter, when most of the ants could be found in the nest bottom (L. Hart and W. R. Tschinkel, unpublished data). This trend suggests that soil and physical conditions affect the characteristics of the nest. The degree to which soil and other abiotic conditions affect ant nest architecture is an unexplored subject.

The nests of *O. brunneus* differ somewhat from those of several other species in being moderately more bottom-heavy than top-heavy. To date, the majority of ant nests are reported to have more chamber area near the surface than near the bottom and chambers closer together near the surface than near the bottom.

A fairly common feature of the nests of *O. brunneus* was their use of cavities made by other animals, including rodents and other ants as well as hollow roots. Such cavities can be recognized because their architecture is very different from that produced by *O. brunneus* excavation. Whether the maker was evicted or had already abandoned the cavity is unknown, but the use of such cavities clearly saves work. This phenomenon has also been observed in other species of ants (unpublished data).

The worker census included only workers that were in the nest at the time the cast was made. Any foragers afield at the time were not included, and their number is unknown.

Filling subterranean ant nests with a casting material can provide more information than just the nest's architecture. It was used to census nests and to determine the distribution of workers within the vertical nest structure. By using paraffin wax to make nest casts, the workers, brood, and alates were fixed at their momentary locations within their ant nests (unpublished data). Melting these casts in sections provides an accurate picture of the distribution of all colony members, brood and food within the vertical nest structure. The recovered ants can also be used for other studies, such as morphometry. Compared to a simple excavation, such casting methods offer the advantage that the casting material finds and fills all the nooks, crannies, and cavities of the nest, capturing all the nest contents in place, something that is difficult to achieve during direct excavation of an uncast nest.

The connection between nest architecture and colony function has received little attention, in part because most studies have been carried out in single-chambered laboratory nests that do not resemble the natural nest. Brian (1956) showed that ants in smaller groups rear brood more efficiently than those in larger groups, a result confirmed by Porter and Tschinkel (1985). Nest architecture combines with the tendency of all ants to sort themselves and their brood to produce social structure within the nest. In most species, workers move centrifugally away from the brood as they (the workers) age (Hölldobler and Wilson 1990; Sendova Franks and Franks 1995), a movement that is connected to age polyethism. In deep nests such as those of the Florida harvester ant, *Pogonomyrmex badius*, and the winter-active ant, *Prenolepis imparts*, this movement sorts workers by age such that the youngest are located mostly in the bottom third of the nest and the oldest (defenders and foragers) near and on the surface (Tschinkel 1987, 1999). In view of the near universality of the centrifugal movement of aging workers away from the brood pile, nest architecture and spatial social structure seems likely to be functional and to contribute to colony fitness. Determining whether these links exist and how they function should be a central question in the study of ant nest architecture.
